# How does school bullying influence adolescent social adaptation? a serial mediation model of school connectedness and self-disclosure

**DOI:** 10.3389/fped.2026.1747055

**Published:** 2026-02-27

**Authors:** Zhe Jin, Jiaxiang Wang, Guoxing Xiang, Pinyi Wang, Ruijin Zhang, Hao Li, Xiong Gan

**Affiliations:** 1 School of Information Management, Central China Normal University, Wuhan, China; 2 Department of Psychology, College of Education and Sports Sciences, Yangtze University, Jingzhou, China

**Keywords:** adolescent, school bullying, school connectedness, self-disclosure, social adaptation

## Abstract

**Introduction:**

School bullying has become an important social problem among adolescents, it can influence the growth of individual, yet understanding of the impacts of school bullying is limited. The present study determined to investigate whether and how school bullying can influence adolescent social adaptation.

**Methods:**

Structural equation modeling was used to assess the hypothesized model. A sample of 434 Chinese adolescents (56.9% females), with an average age of 13.07 years (SD = 0.93), participated the survey.

**Results:**

The present study combined self-disclosure and school connectedness into a serial mediation model, highlighting the role of individual and environmental factors in the outcomes of school bullying.

**Discussion:**

These findings suggest that adolescents who engage in bullying are less likely to disclose personal information, which in turn hinders their sense of belonging at school, ultimately impairing their positive social adaptation. The results highlight the interplay between individual (self-disclosure) and environmental (school connectedness) factors in the outcomes of school bullying. Both limitations and implications are discussed in the end.

## 1 Introduction

Bullying was defined as intentional and repeated aggressive behavior characterized by a power imbalance between perpetrators and victims (1), encompassing physical, verbal, relational, and indirect forms (2). Extensive evidence linked bullying to a broad range of adverse developmental outcomes, including mental and personality disorders, suicidal and violent behaviors, poor physical health, reduced socioeconomic attainment, and diminished subjective well-being (3–8). As adolescents spend substantial time in school, school bullying represents a critical developmental context, with nearly one-third of students reporting peer victimization (9). Although prior research has examined its antecedents and behavioral consequences (10–13), its association with social adaptation—a core indicator of adolescents' socialization and societal integration—remains insufficiently understood.

### 1.1 School bullying and social adaptation

Social adaptation refers to individuals' dynamic adjustment to their social environment (14) and comprises positive and negative dimensions (15). Empirical studies consistently associated school bullying with reduced positive adaptation and increased maladaptation, including lower self-esteem, self-efficacy, active coping, and prosocial behavior, as well as greater social alienation and withdrawal (16–26). From a developmental contextualist perspective, reciprocal person–environment processes suggest that bullying involvement may trigger cumulative maladjustment and long-term behavioral and health risks (27–30). Accordingly, school bullying was hypothesized to negatively predict positive social adaptation and positively predict negative social adaptation (H1).

### 1.2 Mediating effect of school connectedness and self-disclosure

Self-disclosure, defined as revealing personal information to others (31, 32), was central to interpersonal relationships (33). Bullying involvement was associated with reduced self-disclosure, whereas disclosure buffers bullying-related psychological harm and is negatively related to cyberbullying (34–41). Consistent with social penetration theory (42), self-disclosure positively predicts social adaptation across populations (43–49), suggesting a mediating role (H2). School connectedness, reflecting emotional bonds and belonging within school (50), functions as a protective factor (51–53) and mediates the association between bullying and social adaptation (54–59) (H3). Moreover, self-disclosure may promote school connectedness through disclosure reciprocity and enhanced social support (60–64), indicating a serial mediation pathway (H4). Gender and grade were controlled given established differences across study variables (65–72).

### 1.3 The present study

To our knowledge, little research has explored the influence of school bullying on social adaptation and the possible mediating mechanisms underlying this relation. Based on previous evidence and theories, the present study determined to investigate the relationship between school bullying and social adaptation and construct a serial mediation model with hypotheses that:

H1: School bullying can negatively predict positive social adaptation, and positively predict negative social adaptation.

H2: Self-disclosure will mediate the relationship between school bullying and social adaptation.

H3: School connectedness will mediate the relationship between school bullying and social adaptation.

H4: Self-disclosure and school connectedness have a serial mediating effect on the relationship between school bullying and social adaptation.

**Figure 1:**
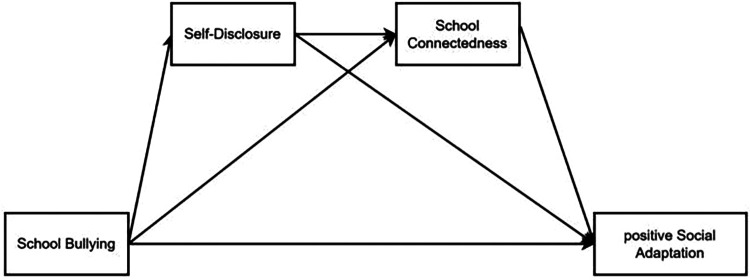
The serial mediation model between SB and PSA. SB, school bullying; PSA, positive social adaptation; SD, self-disclosure; SC, school connectedness.

**Figure 2:**
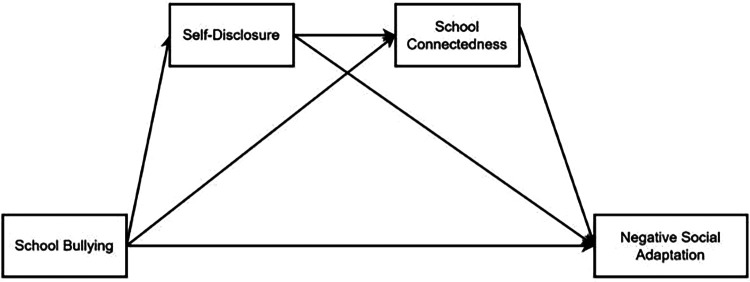
The serial mediation model between SB and NSA. SB, school bullying; NSA, negative social adaptation; SD, self-disclosure; SC, school connectedness.

The following is the hypothesis model diagram of this study (Figures 1, 2).

## 2 Method

### 2.1 Participants and procedure

A total of 434 junior middle school students were recruited from Guizhou province, southwest China. Of these, 247 (56.9%) were females. Their age ranged from 11 to 15 years old, and the average age was 13.07 years (SD = 0.93). This study was approved by the Research Ethics Committee of the Central China Normal University. Before the formal investigation, several junior middle schools were properly negotiated with for the timing of the test administration. We obtained informed consent from participants and their parents or relevant legal guardians and asked them to sign a parental informed consent form. Adolescents were informed that their personal information would be kept confidential, and they had the right to refuse to respond whenever they wanted. The questionnaires of the students who participated voluntarily were collected after they finished, while the remainder were not required to. After obtaining informed consent, students were asked to finish a questionnaire on school bullying, social adaptation, school bonding, self-disclosure, as well as several basic information such as gender and age within half an hour. At the end, researchers collected all the questionnaires and thanked their participation.

### 2.2 Measures

#### 2.2.1 School bullying

Adolescent school bullying was measured by the bully subscale taken from the adapted Chinese version of the Revised Olweus Bully/Victim Questionnaire (ROBVQ; 1, 73). It consists of fourteen items evaluated on a four-point scale (0 = never; 4 = several times a week). One example item is “I posted some students’ secrets, private or embarrassing photos on the Internet.” A higher score indicates more bullying behaviors. This is a one-factor scale.

#### 2.2.2 Social adaptation

Adolescent social adaptation was measured by the Adolescents' Social Adjustment Assessment Scale (ASAAS; 74). This scale has fifty items in total, eight dimensions including self-affirmation, active coping, prosocial tendency, acting efficiency, self-trouble, social alienation, violations and social withdraw. The first four dimensions are considered as the subscale of positive social adaptation, with higher score indicating better positive social adaptation; while other dimensions are considered as the subscale of negative social adaptation, with higher score meaning worse negative social adaptation. One example item is “I am not happy at all.” Responses range from “1 = totally inconsistent” to “5 = totally consistent”.

#### 2.2.3 Self-disclosure

Adolescent self-disclosure was measured by the Adolescent Self-Disclosure with Peers Questionnaire (ASDPQ; 75). This questionnaire includes thirty-seven items, eight dimensions such as interests and study experience. These items were evaluated on a five-point scale (1 = almost nothing; 5 = anything), with a higher score indicating a better level of self-disclosure.

#### 2.2.4 School connectedness

Adolescent school connectedness was measured by the Adolescent School Connectedness Scale (ASCS; 76). It contains ten items. One example is “I feel happy and safe in my school.” Items were rated on a five-point scale ranging from “1 = totally inconsistent” to “5 = totally consistent”. A higher score indicates better connection with school. This is a one-factor scale.

### 2.3 Data analysis

SPSS 23.0 were used in data analyses (77). First, preliminary analyses such as descriptive and correlational statistics were performed in SPSS 23.0 to assess whether the variables were associated with each other. Then, mediation analyses were conducted through PROCESS (Model 6) in SPSS to examine the mediating roles of school connectedness and self-disclosure between school bullying and social adaptation.

### 2.4 Causal relationship statement

It should be noted that the present study employs a cross-sectional design, which precludes the determination of temporal precedence among the study variables. As all measures were collected simultaneously, the observed associations represent contemporaneous relationships rather than directional or causal effects. Therefore, it is not possible to establish whether school bullying precedes changes in self-disclosure, school connectedness, or social adaption, or whether these psychosocial characteristics influence experiences of bullying. Longitudinal designs are needed to more rigorously examine temporal ordering and developmental processes.

## 3 Results

### 3.1 Scale reliability and validity

#### 3.1.1 School bullying

In this study, its Cronbach's alpha coefficient was 0.833.

#### 3.1.2 Social adaptation

In this study, the Cronbach's alpha for positive social adaptation was 0.916, while that for negative social adaptation was 0.893, with the overall Cronbach's alpha coefficient of the scale being 0.703.

#### 3.1.3 Self-disclosure

In this study, the Cronbach's alpha coefficients for these eight dimensions were hobbies 0.819, learning experience 0.850, school situation 0.902, views and attitudes 0.828, physical development 0.936, parent-child relationship 0.877, close friendship 0.934, teacher-student relationship 0.859. Their total Cronbach's alpha coefficient was 0.977 in this study.

#### 3.1.4 School connectedness

In this study, its Cronbach's alpha coefficient was 0.832.

### 3.2 Common method biases analyses

Since the data were from participants' self-report questionnaire, it is necessary to avoid common method biases. And Harman single factor method was recommended to detect it (78). Results showed that there were 39 factors with a characteristic value greater than 1, additionally, the first factor explained a variation of 18.44%, less than the 40% critical value. That is, the results in the present study were less influenced by common method biases.

### 3.3 Descriptive statistics and correlation analyses

First, Pearson correlation analysis was used to explore the association of key variables. As displayed in Table 1, school bullying was negatively associated with positive social adaptation (*r* = −0.279, *p* < 0.01), self-disclosure (*r* = −0.211, *p* < 0.01), school connectedness (*r* = −0.399, *p* < 0.01), while it was positively related with negative social adaptation (*r* = 0.288, *p* < 0.01). Positive social adaptation positively correlated with self-disclosure (*r* = 0.252, *p* < 0.01) and school connectedness (*r* = 0.592, *p* < 0.01), but negatively correlated with negative social adaptation (*r* = −0.357, *p* < 0.01). In addition, negative social adaptation had significantly negative relationships with self-disclosure (*r* = −0.161, *p* < 0.01) and school connectedness (*r* = −0.435, *p* < 0.01). Whereas a significantly positive association was found between self-disclosure and school connectedness (*r* = 0.239, *p* < 0.01).

**Table 1:** Correlation analysis of key variables.

Variable	1	2	3	4	5
1. SB	1				
2. PSA	−0.279**	1			
3. NSA	0.288**	−0.357**	1		
4. SD	−0.211**	0.252**	−0.161**	1	
5. SC	−0.399**	0.592**	−0.435**	0.239**	1

SB, school bullying; PSA, positive social adaptation; NSA, negative social adaptation; SD, self-disclosure; SC, school connectedness.

** *p* < 0.01. The same below.

### 3.4 Mediation effect analyses

#### 3.4.1 Mediating effects between school bullying and positive social adaptation

**Figure 3:**
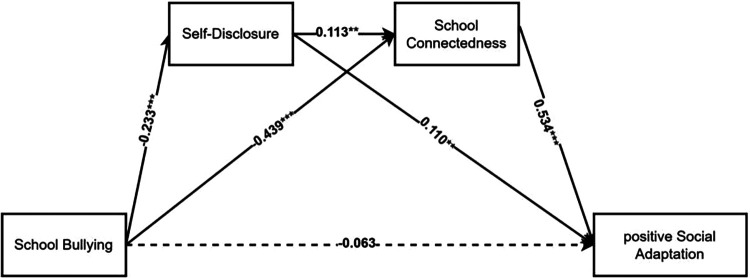
The serial mediation model between SB and PSA. SB, school bullying; PSA, positive social adaptation; SD, self-disclosure; SC, school connectedness. Dashed lines indicate insignificant paths. *p < 0.05; **p < 0.01; ***p < 0.001.

Prior to formal data processing, all variables underwent standardization. This study employed school bullying as the independent variable, positive social adaptation as the dependent variable, and self-disclosure and school connectedness as mediating variables. Using the Bootstrap method with 5,000 repeated sampling iterations to construct a 95% bias-corrected confidence interval, we tested the chain mediating model of “school bullying → self-disclosure → school connectedness → positive social adaptation.” Results with 95% confidence intervals excluding zero indicated statistical significance. As shown in Table 2 and Figure 3, the chain mediating analysis partially replicated the findings of multiple regression analysis. The results of the study indicate that after incorporating the two mediating variables of self-disclosure and school connectedness, school bullying no longer negatively predicts positive social adaptation (*β* = −0.063, *t* = −1.416, *p* = 0.157), indicating that self-disclosure and school connectedness fully mediate the relationship between school bullying and positive social adaptation. Additionally, school bullying negatively predicts self-disclosure (*β* = −0.233, *t* = −4.929, *p* < 0.001) and school connectedness (*β* = −0.439, *t* = −10.122, *p* < 0.001). Self-disclosure exhibits a significant positive predictive effect on school connectedness (*β* = 0.113, *t* = 2.625, *p* < 0.01) and a significant positive predictive effect on positive social adaptation (*β* = 0.110, *t* = 2.744, *p* < 0.01). Finally, school connectedness was found to positively predict positive social adaptation (*β* = 0.534, *t* = 11.970, *p* < 0.001).

**Table 2:** Regression analysis of the serial mediation model between SB and PSA.

Regression equation (*N* = 434)		Fitting index			Significance of coefficient	
Result variable	Predictive variable	*R*	*R* ^2^	*F*	*β*	*t*
PSA		0.368	0.136	22.515***		
	Gender				0.106	1.140
	Grade				−0.235	−2.361*
	SB				−0.337	−7.387***
SD		0.274	0.075	11.662***		
	Gender				0.223	2.326***
	Grade				−0.030	−0.289
	SB				−0.233	−4.929***
SC		0.513	0.263	38.211***		
	Gender				0.199	2.307*
	Grade				−0.125	−1.359
	SB				−0.439	−10.122***
	SD				0.113	2.625**
PSA		0.610	0.373	50.848***		
	Gender				−0.039	−0.481
	Grade				−0.163	−1.916
	SB				−0.063	−1.416
	SD				0.110	2.744**
	SC				0.534	11.970***

SB, school bullying; PSA, positive social adaptation; SD, self-disclosure; SC, school connectedness.

* *p* < 0.05.

** *p* < 0.01.

*** *p* < 0.001.

The chain mediation model identified three indirect pathways, with their effect sizes and 95% confidence intervals presented in Table 3. First, self-disclosure (*β* = −0.026, SE = 0.012, 95% CI [−0.053, −0.006) significantly mediated the relationship between school bullying and positive social adaptation (indirect pathway 1), while school connectedness (*β* = −0.234, SE = 0.038, 95% CI [−0.323, −0.175) played a significant mediating role (indirect pathway 2). Second, self-disclosure and school connectedness (*β* = −0.014, SE = 0.006, 95% CI [−0.029, −0.004) also functioned as chain mediators between school bullying and positive social adaptation (indirect pathway 3). These findings demonstrate that school bullying not only directly impacts positive social adaptation but also indirectly influences adolescents' social well-being through the mediating role of school connectedness and the chain mediation effect of self-disclosure and school connectedness.

**Table 3:** Standardized estimates and 95% CIs for direct and indirect effects.

Path	Standardized estimate	95% CI
Direct effect		
SB → PSA	−0.063	[−0.151, 0.024]
Total indirect effect		
SB → PSA	−0.274	[−0.368, −0.211]
Specific indirect effect		
SB → SD → PSA	−0.026	[−0.053, −0.006]
SB → SC → PSA	−0.234	[−0.323, −0.175]
SB → SD → SC → PSA	−0.014	[−0.029, −0.004]

SB, school bullying; PSA, positive social adaptation; SD, self-disclosure; SC, school connectedness.

#### 3.4.2 Mediating effects between school bullying and negative social adaptation

**Figure 4:**
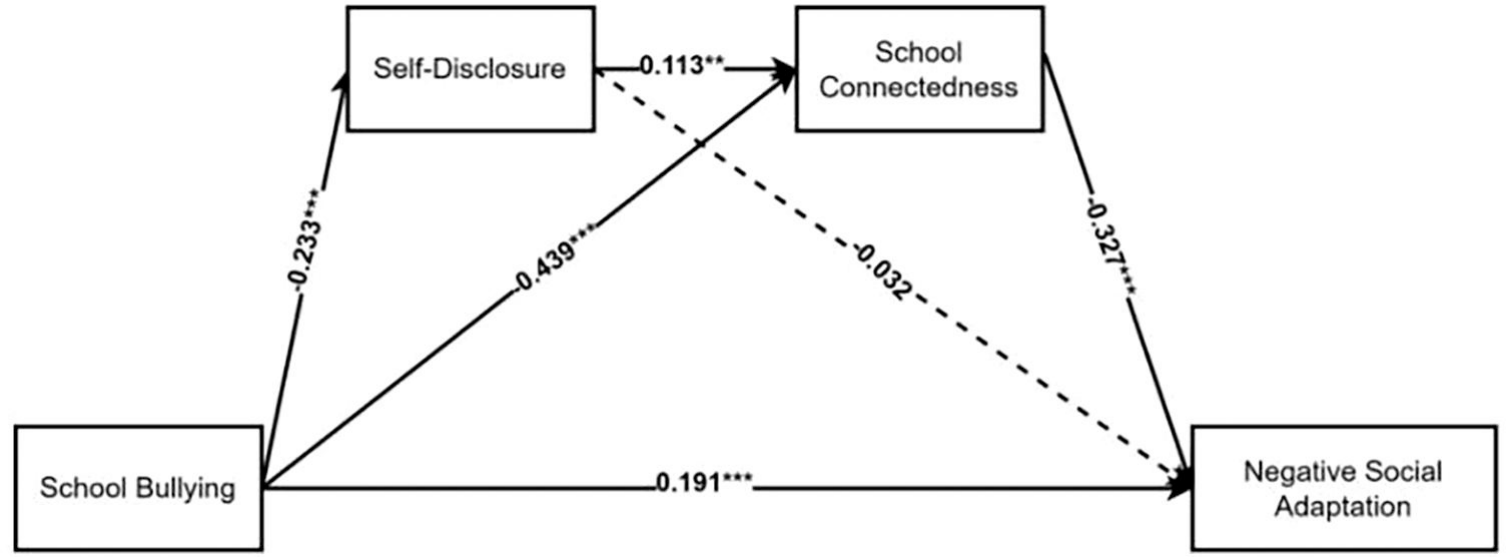
The serial mediation model between SB and NSA. SB, school bullying; NSA, negative social adaptation; SD, self-disclosure; SC, school connectedness. Dashed lines indicate insignificant paths. *p < 0.05; **p < 0.01; ***p < 0.001.

Likewise, another serial mediation model was also constructed to test the possible influence of self-disclosure and school connectedness on the association between school bullying and negative social adaptation. As shown in Table 4 and Figure 4, school bullying positively predicts negative social adaptation (*β* = 0.191, *t* = 3.872, *p* < 0.001), indicating that self-disclosure and school connectedness serve as chain mediators between school bullying and negative social adaptation. Additionally, school bullying negatively predicts self-disclosure (*β* = −0.233, *t* = −4.929, *p* < 0.001) and school connectedness (*β* = −0.439, *t* = −10.122, *p* < 0.001). Self-disclosure significantly and positively predicts school connectedness (*β* = 0.113, *t* = 2.625, *p* < 0.01), but does not exhibit a negative predictive effect on negative social adaptation (*β* = −0.032, *t* = −0.726, *p* = 0.469). Finally, school connectedness were found to negatively predict negative social adaptation (*β* = −0.327, *t* = −6.614, *p* < 0.001).

**Table 4:** Regression analysis of the serial mediation model between SB and NSA.

Regression equation (*N* = 434)		Fitting index			Significance of coefficient	
Result variable	Predictive variable	*R*	*R* ^2^	*F*	*β*	*t*
NSA		0.384	0.147	24.743***		
	Gender				−0.095	−1.034
	Grade				0.259	2.625*
	SB				0.351	7.737***
SD		0.274	0.075	11.662***		
	Gender				0.223	2.326***
	Grade				−0.030	−0.289
	SB				−0.233	−4.929***
SC		0.513	0.263	38.211***		
	Gender				0.199	2.307*
	Grade				−0.125	−1.359
	SB				−0.439	−10.122***
	SD				0.113	2.625**
NSA		0.480	0.230	25.613***		
	Gender				−0.015	−0.166
	Grade				0.216	2.292*
	SB				0.191	3.872***
	SD				−0.032	−0.726
	SC				−0.327	−6.614***

SB, school bullying; NSA, negative social adaptation; SD, self-disclosure; SC, school connectedness.

* *p* < 0.05.

** *p* < 0.01.

*** *p* < 0.001.

The chain mediation model reveals three indirect pathways, with their effect sizes and 95% confidence intervals presented in Table 5. First, self-disclosure (*β* = 0.008, SE = 0.011, 95% CI [−0.015, 0.029) significantly mediates the relationship between school bullying and negative social adaptation (indirect pathway 1). Second, self-disclosure and school connectedness (*β* = 0.009, SE = 0.004, 95% CI [0.002, 0.019) also mediate this relationship through a chain effect (indirect pathway 2). These findings demonstrate that school bullying not only directly impacts negative social adaptation but also indirectly influences adolescents' negative social adaptation via the mediating role of school connectedness and the chain mediation effect of self-disclosure and school connectedness.

**Table 5:** Standardized estimates and 95% CIs for direct and indirect effects.

Path	Standardized estimate	95% CI
Direct effect		
SB → NSA	0.191	[0.094, 0.288]
Total indirect effect		
SB → NSA	0.159	[0.095, 0.246]
Specific indirect effect		
SB → SC → NSA	0.143	[0.086, 0.228]
SB → SD → SC → NSA	0.009	[0.002, 0.019]

SB, school bullying; NSA, negative social adaptation; SD, self-disclosure; SC, school connectedness.

## 4 Discussion

The present study attempts to figure out the direct and indirect relationships between school bullying and social adaptation during adolescence by simultaneously assess the mediation effects of self-disclosure and school connectedness in a serial mediation model. Structural equation modeling was performed to analyze data from a sample of Chinese students, and the findings generally supported the hypothesized model. School bullying had influence on the social adaptation through the mediating pathways of self-disclosure and school connectedness.

To start, the first hypothesis about the direct relationship between school bullying and social adaptation was totally verified. Specifically, school bullying could negatively predict positive social adaptation, while it could positively predict negative social adaptation. That is, compared with adolescents without experiencing school bullying, those who are involved in bullying others are more likely to develop less positive social adaptation outcomes and more negative social adaptation outcomes. This finding is consistent with previous research, which found a significant relationship between school bullying and specific content of social adaptation (e.g., self-affirmation, prosocial behavior and social alienation (19, 22, 23, 78). Moreover, this finding repeatedly confirmed the opinion of Olweus (79) that bullying reflects a stable aggressive, antisocial and rule-breaking personality, which makes children prone to social maladaptation in adolescence.

One possible explanation of this finding given by developmental contextualism is that individuals have impacts on their environment, meanwhile, individual development will also be influenced by environment (27). Adolescent bullying others will bring negative impacts to victims, such as physical injury and feeling of insecurity. In the meantime, their peers will keep a wide berth of them and not be willing to develop friendship with them, because bullying is considered as a deviant behavior in violation of the moral rules. As a result, those who bully others will be punished, excluded and lose friendship. More seriously, they will develop cognitive bias of this kind of deviant behavior, believing that bullying others is a symbol of power, which is obviously harmful. In a long term, they cannot adapt to the complex society, and develop more problems including theft, violence, binge-drinking and smoking (29, 30).

The present study revealed that self-disclosure could mediate the relationship between school bullying and positive social adaptation, rather than negative social adaptation. This is similar to Adams and Cantin's (37) the second hypothesis partly received support. We believe the reason why self-disclosure fails to mediate the relationship between school bullying and negative social adaptation is adolescent involved in school bullying will less likely to disclose themselves, which is not beneficial to positive social adaptation. This finding integrates many previous studies that self-disclosure is closely associated with school bullying and social adaptation respectively among adolescents (35, 36, 47, 48).

The social penetration theory thinks that self-disclosure is of vital importance in the development of interpersonal relationships and social adaptation (42). Because self-disclose can contribute to information exchange, making oneself understood by others, and then others will respond to the information. The feedback can help oneself to keep or correct behaviors. This process will be beneficial to individual growth, because adolescents can feel being supported, cared and encouraged by obtaining feedback, especially when they get positive feedback. In a long term, adolescents will be brave to ask for advice or feedback when they are facing difficulties, they will cope with positive ways and take action efficiently. More importantly, they will develop affirmation and confidence in themselves. All of these outcomes could promote positive social adaptation. Interestingly, although self-disclosure was negatively related to negative social adaptation and positively related to school bullying, it could not mediate the association between them. One possible reason is the masking effect of the direct pathway. Compared with the association between school bullying and positive social adaptation, school bullying and negative social adaptation are both negative developmental problems, so they are more easily to affect each other directly.

In addition, school connectedness is another mediator between school bullying and social adaptation, which is in favor of the third hypothesis. Experiencing bullying others at school will make adolescents disconnect with their schools, which will lead to less positive social adaptation and more negative social adaptation. This finding is similar to Gao et al.'s finding (59) and consistent with prior studies that school connectedness was closely associated with adolescent school bullying and social adaptation outcomes (60, 61).

Furthermore, in combination with social identity theory and Maslow's hierarchy of needs theory could provide a better understanding of this finding (80, 81). The membership of social groups is an important part of self-concept. In social interaction, people always strive to maintain positive social identity to enhance the sense of self-worth. Additionally, individual have a need for belongingness and love. Thereby, in order to seek for membership, sense of belonging and love, adolescents will spare no effects to build association with their school, teachers and peers. As mentioned before, building relationship at school is a rehearsal and preparation to build connectedness with the real world. A well-prepared individual is more likely to adapt to the complex society (57). However, experiencing bullying at school will make adolescents feel insecure, competitive, even dangerous, so it is very difficult for them to connect with school, and they will not feel supported, recognized and valuable. As a result, it is too hard for them to positively adapt to the social environment.

Another important finding of the present study is that self-disclosure and school connectedness serve as serial mediators between school bullying and social adaptation during adolescence, which supports the fourth hypothesis. In other words, experiencing school bullying will make adolescents not willing to disclose themselves, sequentially, they might not build connection with their school, which will influence their adaptation to society. Similarly, in combination with the disclosure reciprocity effect and social penetration theory could also help better understanding this finding (42, 60). Knowing each other is a prerequisite for establishing a relationship. Self-disclosure will let others know oneself, simultaneously, others will also respond to disclose themselves. Thus, they know each other more deeply, and gradually develop a relationship, which will make them feel supported, cared and encouraged at school. As a result, they will connect with their schools and peers. However, engagement in school bullying will reduce the possibility to share private information with others, so they will not build school connectedness, which will have impacts on their adaptation to society. This finding extends prior studies by figuring out that self-disclosure and school connectedness could have a sequential mediating effect on the relationship between school bullying and social adaptation.

Despite its unique advantages, the present study is not without limitations. First, with regard to participants, the present study has only recruited 434 Chinese junior middle school students, which is not a large sample. And the findings cannot be generalized to other countries. So future studies can include more participants from different culture backgrounds. Second, all the data are collected by self-reported questionnaires, which cannot avoid the potential bias. Therefore, future studies can use other ways to collect data, such as combining other-reported questionnaires and other indicators. Third, the present study used a cross-sectional design and cannot obtain causal results, but adolescent development is a dynamic process, future studies should use a longitudinal design to further explore the relationship between school bullying and social adaptation. Forth, the present study did not involve any moderators. Future studies should attempt to explore whether the relationship between school bullying and social adaptation is stable in different situations.

By constructing a serial mediation model, the present study demonstrated that school bullying had influence on adolescent social adaptation directly, as well as indirectly through the serial mediating mechanisms of self-disclosure and school connectedness. These findings highlight the importance of simultaneously examining self-disclosure and school connectedness as mediators between school bullying and adolescent social adaptation. Regarding interventions aimed to prevent or reduce adolescent school bullying, practitioners should pay more attention to the combination of individual and environment factors.

## Data Availability

The raw data supporting the conclusions of this article will be made available by the authors, without undue reservation.
